# Kaempferol Ameliorates Non-Alcoholic Fatty Liver Disease by Targeting TRIM56 to Regulate Lipid Metabolism

**DOI:** 10.3390/ijms27093767

**Published:** 2026-04-23

**Authors:** Hui Yang, Yingrui Wang, Lejian Zhu, Zhuoxi Chen, Shuo Yan, Yuan Du, Binsheng Wang, Leiming Zhang

**Affiliations:** 1 School of Traditional Chinese Medicine, Shandong Medical and Pharmaceutical University, Yantai 264003, China; yangh2527@163.com (H.Y.);; 2 Key Laboratory of Molecular Pharmacology and Drug Evaluation, Ministry of Education, School of Pharmacy, Yantai University, Yantai 264005, China

**Keywords:** TRIM56, non-alcoholic fatty liver disease, kaempferol, lipid metabolism, fatty acid synthase

## Abstract

Non-alcoholic fatty liver disease (NAFLD) is characterized by excessive hepatic lipid accumulation and can progress to severe liver injury. Kaempferol (KPF), a plant-derived flavonoid, exhibits lipid-regulatory properties. Tripartite motif-containing protein 56 (TRIM56), an E3 ubiquitin ligase, has been reported to interact with fatty acid synthase (FASN) and limit hepatic lipogenesis. This study investigated whether KPF alleviates NAFLD through modulation of TRIM56-associated lipid metabolic pathways. Molecular docking, molecular dynamics simulations, and cellular thermal shift assays (CETSA) were employed to evaluate the interaction between KPF and TRIM56. High-fat diet-induced NAFLD mice and fatty acid-treated HepG2 cells were used to assess the effects of KPF on hepatic lipid accumulation. Histological analysis, lipid profiling, Oil Red O staining, Western blotting, immunofluorescence, and quantitative PCR were performed. Endogenous co-immunoprecipitation examined the association between TRIM56 and FASN, and siRNA-mediated knockdown of TRIM56 evaluated its functional contribution. KPF significantly reduced serum triglyceride, total cholesterol, and low-density lipoprotein cholesterol levels, ameliorated hepatic steatosis in vivo, and decreased intracellular lipid accumulation in vitro. In silico and CETSA analyses supported the engagement of TRIM56 by KPF. KPF restored TRIM56 expression under steatotic conditions, whereas TRIM56 silencing attenuated its lipid-lowering effects. TRIM56 was confirmed to associate with FASN, and KPF treatment suppressed multiple lipogenic enzymes. These findings indicate that KPF alleviates hepatic steatosis, at least in part, through modulation of TRIM56-associated lipogenic pathways, highlighting TRIM56 as a potential therapeutic target in NAFLD.

## 1. Introduction

Non-alcoholic fatty liver disease (NAFLD) denotes liver obesity due to the absence of substantial alcohol intake and is the most widespread chronic liver ailment globally [1]. Surveys in epidemiology show that roughly a quarter to a third of people worldwide are impacted by NAFLD [2]. This condition is prevalent in those dealing with obesity, type 2 diabetes, or metabolic syndrome. NAFLD is not a one-size-fits-all issue; it is more of a broad range of diseases. It starts with simple fat and goes all the way up to a more serious condition known as non-alcoholic steatohepatitis (NASH). This is a step-up from the first stage and is marked by liver cell damage, inflammation in the lobules, and different levels of scarring [3]. As time goes on, NASH can evolve into severe liver complications such as cirrhosis, hepatic decompensation, and hepatocellular carcinoma (HCC), making it a significant menace to worldwide health and adding to the growing financial strain on healthcare systems [4,5,6]. Although NAFLD is increasingly recognized as a major cause of chronic liver disease, no approved pharmacological treatment exists. Current management depends mainly on long-term, hard-to-sustain lifestyle changes and weight loss. Several drug candidates, including PPAR/FXR agonists and GLP-1 analogues, are in clinical trials, but their efficacy and safety are still limited [7,8,9]. This therapeutic gap highlights the urgent need for novel agents that can target the underlying molecular drivers of NAFLD.

The primary pathology of NAFLD lies in disrupted hepatic lipid homeostasis. This manifests as increased de novo lipogenesis, impaired fatty acid β-oxidation, and diminished VLDL secretion, resulting in excessive triglyceride accumulation, lipotoxicity, oxidative stress, and chronic metabolic inflammation [10,11]. Considering these interconnected mechanisms, targeting hepatic lipid metabolism has emerged as an attractive therapeutic approach [12]. Bioactive natural compounds, including flavonoids, have received considerable attention due to their wide-ranging pharmacological properties, which include anti-inflammatory, antioxidant, insulin-sensitizing, and lipid-lowering effects [13,14]. Among these compounds, kaempferol (KPF), a flavonoid found abundantly in fruits, vegetables, and medicinal plants [15], has potent biological activities such as reducing inflammatory responses, suppressing oxidative injury, exerting anti-tumor effects, and providing neuroprotection [16,17]. KPF has also been associated with improving metabolic dysfunction by modulating signaling pathways involved in lipid regulation and oxidative stress responses [18,19]. However, the exact molecular targets responsible for its liver-protective effects in NAFLD remain poorly understood.

Emerging research has identified tripartite motif-containing protein 56 (TRIM56), an E3 ubiquitin ligase, as a key regulator of hepatic lipid metabolism [20]. TRIM56 levels are significantly decreased in NAFLD, and hepatocyte-specific upregulation of this enzyme has been shown to attenuate steatosis by promoting the ubiquitination and subsequent degradation of fatty acid synthase (FASN), a major lipogenic enzyme responsible for fatty acid synthesis [21,22]. These discoveries position TRIM56 as a central molecular regulator of liver lipid homeostasis and suggest its potential as a novel therapeutic target for fatty liver disease.

In this study, we investigated whether KPF alleviates hepatic steatosis by targeting TRIM56 and modulating lipid metabolic pathways. Using in vivo and in vitro models of NAFLD, combined with computational prediction, biochemical validation, and functional perturbation of TRIM56, aiming to clarify the role of TRIM56 in mediating the KPF anti-fatty degeneration process, this study demonstrates that TRIM56 may become an important potential target for KPF regulation of NAFLD lipid metabolism.

## 2. Results

### 2.1. KPF Reduces Serum Lipid Levels and Improves Liver Histology in Mice with NAFLD

**Figure 1:**
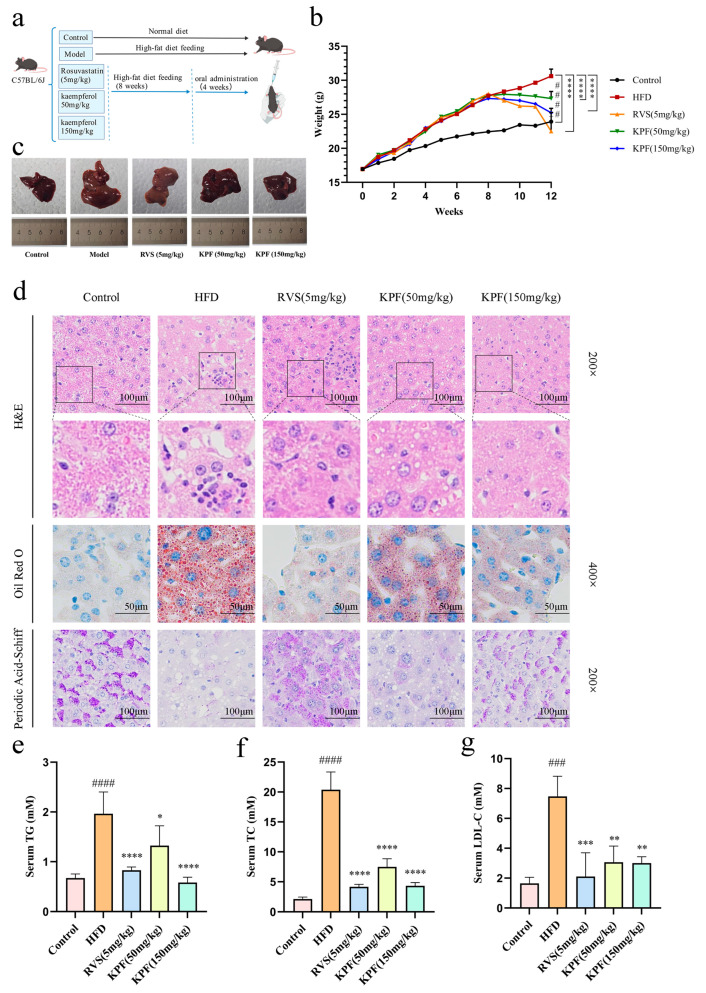
KPF reduces serum lipid levels and improves liver histology in mice with NAFLD: (**a**) The process of in vivo experimental animal model induction. (**b**) Changes in mouse body weight after 8 weeks of high-fat diet induction of a non-alcoholic fatty liver disease model and 4 weeks of KPF gavage administration. (**c**) The effect of KPF on liver size in mouse models of NAFLD induced by a high-fat diet. (**d**) Hematoxylin and eosin (H&E) staining of mouse liver (200×), Oil Red O staining of mouse liver (400×), and PAS staining of mouse liver (200×). (**e**–**g**) Detection of serum TG, TC, and LDL-C in mice. All data are presented as mean ± SD. For serum lipid measurements, *n* = 3 biologically independent mice per group. Statistical significance was analyzed by one-way ANOVA with Tukey’s post hoc test, except for body weight changes, which were analyzed by two-way repeated-measures ANOVA. Compared with the control group, ^###^ *p* < 0.001, ^####^ *p* < 0.0001; compared with the model group, * *p* < 0.05, ** *p* < 0.01, *** *p* < 0.001, **** *p* < 0.0001.

The therapeutic potential of KPF against diet-induced hepatic steatosis was evaluated in a mouse model of NAFLD induced by prolonged exposure to an HFD (high-fat diet). After an initial 8-week HFD feeding period, mice were treated for 4 weeks (oral treatment phase) to evaluate the pharmacological effects of KPF (Figure 1a). Consistent with NAFLD progression, animals on an HFD showed a continuous increase in body weight throughout the modeling period. KPF-treated animals showed a gradual decrease in body mass after compound administration. The most significant decline was observed in the high-dose KPF group, where the trend closely matched that of the rosuvastatin-treated positive control group. At week 12, there were statistically significant differences in body weight among the groups of mice (*p* < 0.0001) (Figure 1b). Gross morphological study of hepatic tissues further supported the positive effect of KPF. Livers collected from HFD-fed mice appeared significantly enlarged and pale, typical of severe steatosis. In comparison, hepatic size in animals given low or high doses of KPF decreased gradually, indicating reduced HFD-induced hepatomegaly. However, gross morphology serves only as a supplementary observation criterion; the assessment of treatment efficacy is still primarily based on histological and biochemical findings (Figure 1c). Histopathological examination provided further confirmation of KPF’s hepatoprotective effects. H&E staining showed that the HFD group was characterized primarily by hepatic steatosis, with focal inflammatory changes also observed in some areas, reflecting advanced NAFLD pathology. These abnormalities were substantially ameliorated after KPF treatment. The high-dose KPF group showed nearly normal hepatic architecture, with significantly less steatosis and inflammation. Lipid deposition was further evaluated using Oil Red O staining, which showed extensive intracellular lipid accumulation in hepatocytes of HFD-fed animals. KPF administration reduced the number of lipid droplets in a dose-dependent manner, demonstrating its strong ability to lower lipids. PAS staining revealed severe impairment of hepatic glycogen storage in HFD mice, while KPF treatment restored glycogen levels, indicating improved metabolic function in hepatocytes (Figure 1d).

Serum biochemical parameters confirmed the histological findings. Mice on an HFD showed significantly higher circulating lipid levels compared to those on a standard diet. Four weeks of KPF treatment significantly improved systemic lipid metabolism. HFD caused significant increases in serum triglycerides (TG), total cholesterol (TC), and low-density lipoprotein cholesterol (LDL-C). KPF reversed these changes in a dose-dependent manner. At 150 mg/kg, KPF lowered TG by about 48%, TC by 42%, and LDL-C by 50%, closely approximating the effects of rosuvastatin (5 mg/kg) (*p* < 0.0001, *p* = 0.0035). Furthermore, there was no statistically significant difference between the two groups (Figure 1e–g). Rosuvastatin, used as a positive control, also markedly improved HFD-induced hepatic steatosis and dyslipidemia, as evidenced by reduced liver enlargement, improved histological appearance, and lower serum TG, TC, and LDL-C levels. These data support the responsiveness of the animal model to a known lipid-lowering intervention.

These results establish the lipid-lowering and hepatoprotective effects of KPF in vivo, providing a phenotypic basis for subsequent mechanistic investigations.

### 2.2. KPF Reduces Lipid Accumulation in HepG2 Cells

**Figure 2:**
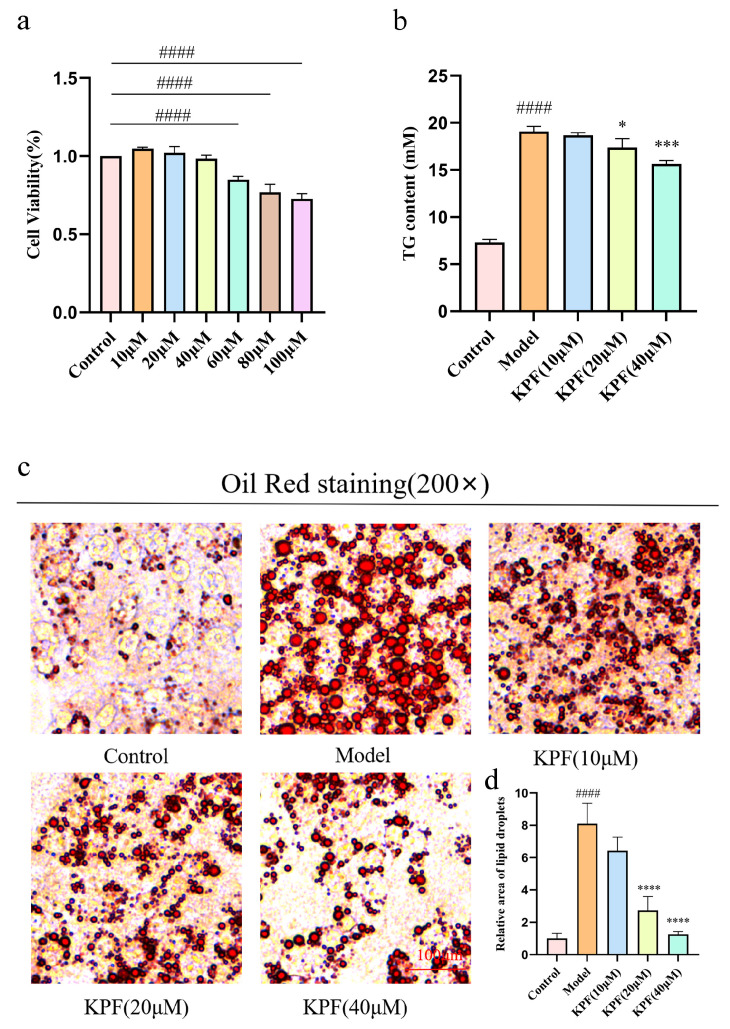
KPF reduces lipid accumulation in HepG2 cells: (**a**) CCK8 assay for the effect of different concentrations of KPF on HepG2 cell viability. (**b**) Detection of TG in cell supernatant. (**c**,**d**) Oil Red O staining to observe lipid droplet formation in cells induced with the palmitate/oleate mixture for 24–48 h and subsequently treated with different concentrations of KPF for 24 h. All data are presented as mean ± SD from *n* = 3 independent biological experiments. Compared with control, ^####^ *p* < 0.0001; compared with model, * *p* < 0.05, *** *p* < 0.001, **** *p* < 0.0001.

To explore the direct cellular effects of KPF on lipid accumulation, its cytotoxicity toward HepG2 hepatocytes was initially evaluated using the CCK-8 assay. Concentrations of 10 μM, 20 μM, and 40 μM were identified as non-cytotoxic and were selected for further experiments (*p* < 0.0001) (Figure 2a). Intracellular lipid accumulation was induced by exposing HepG2 cells to a 2:1 mixture of palmitic acid and oleic acid, resulting in a final concentration of 1 mM. Measurement of TGs in the culture supernatant showed that fatty acid treatment significantly increased TG levels compared with untreated controls. KPF treatment effectively reduced this increase, leading to a significant reduction in TG content (*p* = 0.0235, *p* = 0.0001) (Figure 2b). Lipid droplet accumulation was further investigated using Oil Red O staining. Control cells showed minimal staining, whereas fatty acid-treated HepG2 cells showed substantial lipid droplet accumulation, consistent with intracellular steatosis. Treatment with KPF significantly reduced lipid droplet formation. The reduction was particularly observed at 20 μM and 40 μM, where positively stained areas decreased sharply compared to the steatosis model group (*p* < 0.0001) (Figure 2c,d). These observations indicate that KPF directly modulates hepatocellular lipid metabolism under steatotic stress, suggesting the involvement of intracellular regulatory pathways.

### 2.3. Investigation of the Mechanism by Which KPF Regulates TRIM56

**Figure 3:**
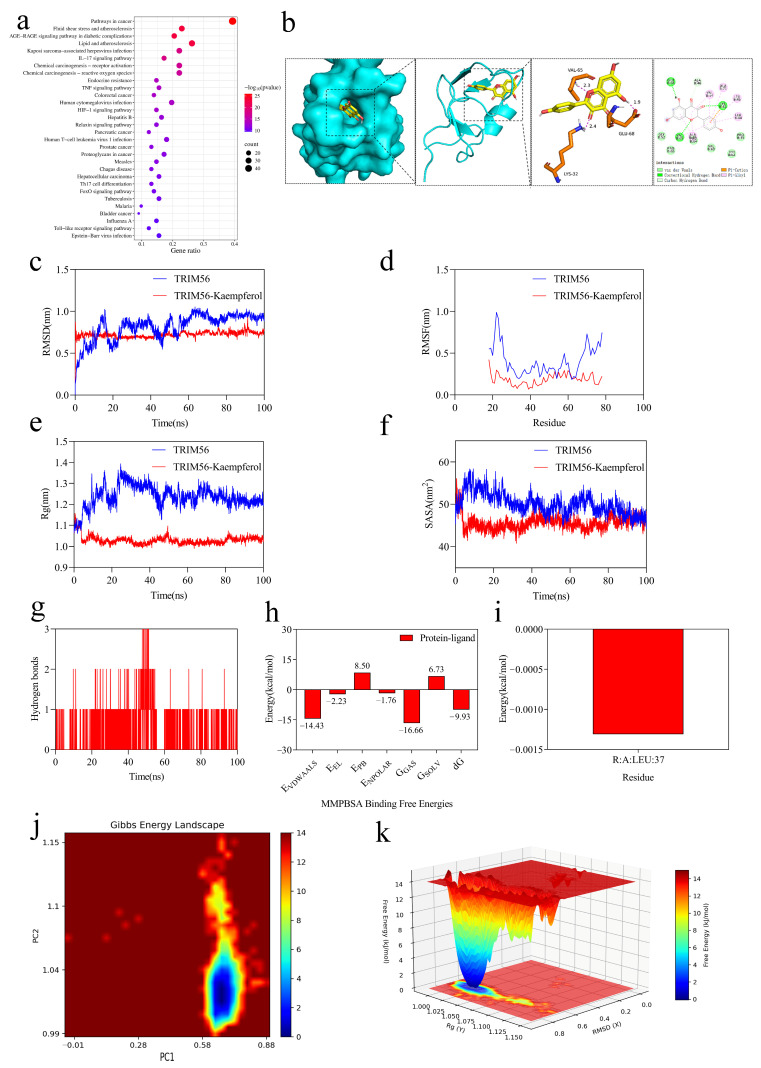
Computational analysis of the interaction between KPF and TRIM56: (**a**) KEGG pathway enrichment analysis of overlapping targets between KPF and NAFLD-related genes. (**b**) Molecular docking model of KPF with TRIM56. (**c**) RMSD of protein and protein–ligand complex during molecular dynamics simulation. (**d**) RMSF of TRIM56 residues. (**e**) Radius of gyration (Rg) of TRIM56 and the TRIM56–KPF complex. (**f**) Solvent-accessible surface area (SASA) during simulation. (**g**) Number of hydrogen bonds formed between KPF and TRIM56. (**h**) Binding free energy profile of the TRIM56–KPF complex. (**i**) Per-residue energy decomposition of TRIM56. (**j**,**k**) Gibbs free energy landscape analysis of the TRIM56–KPF system.

To explore the molecular mechanism underlying the lipid-lowering effects of KPF, we first investigated whether TRIM56 serves as a direct molecular target of KPF in NAFLD models. Network pharmacology was used to identify potential molecular targets of KPF and to identify overlaps between KPF-associated targets and NAFLD-related genes. KEGG pathway enrichment analysis revealed that the overlapping targets were predominantly associated with lipid metabolism, atherosclerosis, and related metabolic pathways (Figure 3a). TRIM56 has been previously identified as a negative regulator of hepatic steatosis by binding to and degrading FASN, thus limiting de novo lipogenesis. Based on this functional significance, it was hypothesized that KPF may modulate NAFLD progression by regulating TRIM56 [21].

**Figure 4:**
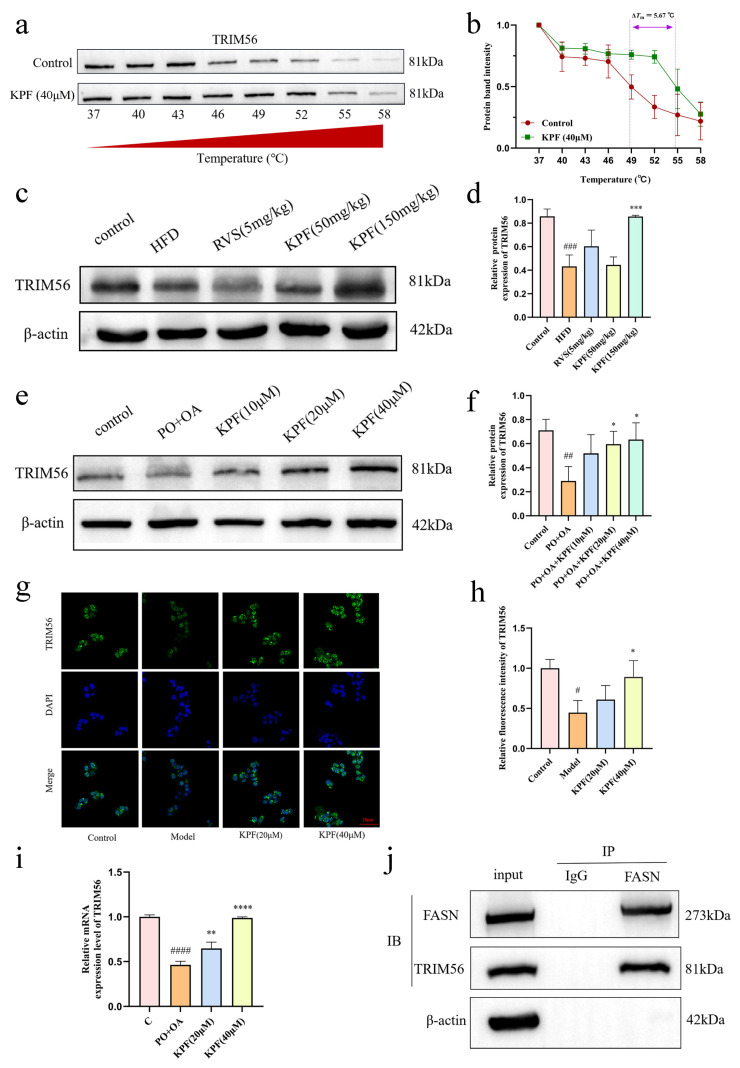
KPF regulates TRIM56 expression and associates with FASN: (**a**,**b**) Cellular thermal shift assay (CETSA) analysis of TRIM56 stability in HepG2 cells treated with KPF (*n* = 3). (**c**–**f**) Western blot analysis of TRIM56 expression in liver tissues and HepG2 cells (*n* = 3). (**g**,**h**) Immunofluorescence staining showing TRIM56 expression in HepG2 cells (*n* = 3). (**i**) Quantitative PCR analysis of TRIM56 mRNA expression (*n* = 3). (**j**) Endogenous co-immunoprecipitation showing the association between TRIM56 and FASN. All results are represented as means ± SD. Compared with control, ^#^ *p* < 0.05, ^##^ *p* < 0.01, ^###^ *p* < 0.001, ^####^ *p* < 0.0001; compared with model, * *p* < 0.05, ** *p* < 0.01, *** *p* < 0.001, **** *p* < 0.0001.

To investigate this potential interaction, molecular docking was employed. Docking simulations showed that KPF forms a stable interaction with the GLU-68 residue of TRIM56, involving a hydrogen bond measuring 1.9 Å. Molecular docking predicted that KPF could interact with TRIM56 with a binding energy of −7.0 kcal/mol, suggesting a favorable and potentially stable interaction. However, this docking score should be interpreted as a relative in silico estimate rather than a direct experimental measurement of binding affinity (Figure 3b). The molecular dynamics simulation results indicate that KPF and TRIM56 reached a stable state after 10 ns (Figure 3c,d). TRIM56 became more tightly folded and increased in stability after binding to KPF (Figure 3e,f). The ligand stably bound to the site without significant movement. Hydrogen bond interactions contributed to the stable binding (Figure 3g). The energy decomposition analysis showed that the binding free energy between them was −9.93 kcal/mol during the 0–100 ns period, indicating very stable binding, with LEU:37 being the major contributing residue (Figure 3h,i). Both free energy maps reveal a distinct deep blue energy trough in the TRIM56–KPF complex, corresponding to a high-probability stable conformational cluster in the system. This indicates that the bound state is thermodynamically stable, and the clear separation between the energy plateau and the energy barrier distinguishes the stable conformation from other conformations, suggesting that conformational transitions in the complex are subject to a kinetic bottleneck, resulting in a highly stable overall binding mode (Figure 3j,k). These data collectively suggest that the binding of KPF to TRIM56 is stable in molecular dynamics simulations and may have biological activity. CETSA was used to evaluate target engagement by detecting changes in the receptor protein’s thermal stability after exposure to the small molecule [23]. HepG2 cells treated with 40 μM KPF for 3 h showed increased thermal stability of TRIM56, indicated by less temperature-induced degradation compared to untreated controls. The melting temperature (Tm) of the target protein was calculated by four-parameter logistic (4PL) curve fitting. The Tm of the control group was 48.99 °C, while the Tm of the 40 μM KPF group was 54.66 °C, indicating that KPF treatment significantly increased the thermal stability of the protein. (Figure 4a,b). These results indicate that KPF engages TRIM56 and enhances its protein stability, supporting a functional interaction that may contribute to the lipid-regulatory effects of KPF.

Western blot analysis further supported this relationship. In hepatic tissues from NAFLD mice and fatty acid-treated HepG2 cells, TRIM56 expression was significantly decreased (*p* = 0.0009, *p* = 0.002). KPF (150 mg/kg) significantly promotes the expression of TRIM56 in mouse liver tissue (*p* = 0.0009). In vitro experiments have also demonstrated that kaempferol promotes the expression of TRIM56 in HepG2 cells in a dose-dependent manner (*p* = 0.0248, *p* = 0.0106) (Figure 4c–f). Immunofluorescence staining also showed a significant upregulation of TRIM56 after KPF treatment at 20 μM and 40 μM, with increases proportional to the dose (*p* = 0.0136, *p* = 0.041) (Figure 4g,h). Transcriptional analysis using qPCR confirmed that KPF at 20 μM and 40 μM significantly increased TRIM56 mRNA levels in HepG2 cells (*p* < 0.0001, *p* = 0.0029) (Figure 4i).

Given previous reports indicating that TRIM56 can interact with FASN [21], we next examined whether TRIM56 associates with FASN in our experimental system. Endogenous co-immunoprecipitation assays were performed using an anti-FASN antibody; TRIM56 was readily detected in the FASN immunoprecipitates, whereas no obvious signal was observed in the IgG control (Figure 4j). These results demonstrate that TRIM56 and FASN form a protein complex under basal conditions, supporting the existence of a TRIM56–FASN regulatory axis relevant to lipid metabolism.

Collectively, these results demonstrate that KPF engages TRIM56 and restores its reduced expression under steatotic conditions. In addition, endogenous co-immunoprecipitation confirms that TRIM56 associates with FASN in hepatocytes, supporting the presence of a TRIM56–FASN protein complex relevant to lipid metabolism. These findings provide a molecular basis for further investigating the role of TRIM56 in mediating the downstream lipid-regulatory effects of KPF.

### 2.4. KPF Regulates Lipid Synthesis-Related Proteins Downstream of TRIM56

**Figure 5:**
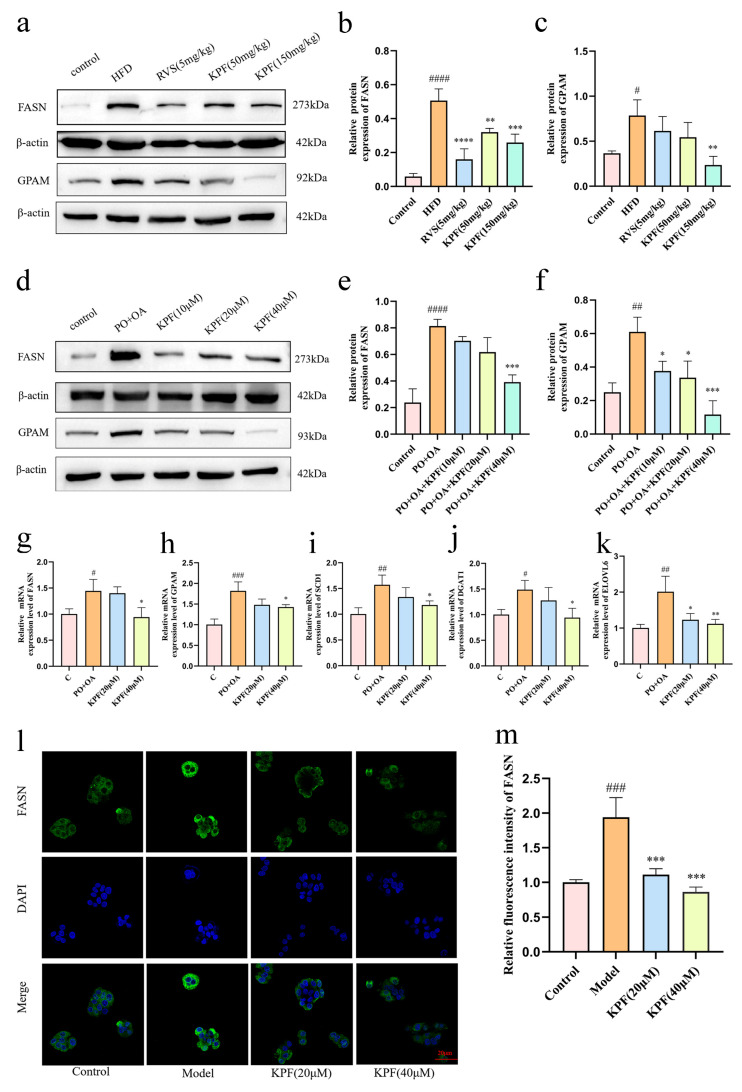
KPF alters the expression of lipogenic enzymes associated with TRIM56: (**a**–**f**) Western blot detection of the expression changes of FASN and GPAM, key lipid metabolism synthases downstream of TRIM56, in in vivo and in vitro models. (**g**–**k**) qPCR analysis of the mRNA levels of lipid metabolism-related synthases at the transcriptional level (*n* = 3). (**l**,**m**) Immunofluorescence-assisted detection of the expression of the key target FASN (*n* = 3). All results are represented as means ± SD. Compared with control, ^#^ *p* < 0.05, ^##^ *p* < 0.01, ^###^ *p* < 0.001, ^####^ *p* < 0.0001; compared with model, * *p* < 0.05, ** *p* < 0.01, *** *p* < 0.001, **** *p* < 0.0001.

Given the association between TRIM56 and FASN, we next examined whether KPF affects the expression of key enzymes involved in hepatic lipogenesis. Western blot results showed that FASN and glycerol-3-phosphate acyltransferase (GPAM), two crucial enzymes involved in fatty acid elongation and triglyceride biosynthesis, were significantly upregulated in both HFD-induced NAFLD mouse livers and fatty acid-loaded HepG2 cells (*p* < 0.0001, *p* = 0.0231, *p* = 0.0016). Treatment with KPF significantly reduced the protein levels of FASN and GPAM in mouse livers, indicating a significant decrease in lipid synthesis activity (*p* = 0.0057, *p* = 0.0007). In vitro experiments also demonstrated the same effect of KPF (*p* = 0.0004, *p* = 0.0288, *p* = 0.0111, *p* = 0.0001) (Figure 5a–f).

Transcriptional profiling via qPCR further supported these findings, showing that KPF decreased mRNA levels of key lipogenic genes, including *FASN* (*p* = 0.0222), *GPAM* (*p* = 0.0479), diacylglycerol acyltransferase (*DGAT*) (*p* = 0.0205), stearoyl-CoA desaturase 1 (*SCD1*) (*p* = 0.0493), and very long-chain fatty acid elongase 6 (*ELOVL6*) (*p* = 0.0192, 0.0092). These genes are crucial for fatty acid elongation, desaturation, and triglyceride synthesis. Their synchronized reduction suggests that KPF exerts broad inhibitory effects on hepatic lipogenesis (Figure 5g–k).

Consistent with these results, immunofluorescence staining confirmed the molecular findings. Lipid-loaded HepG2 cells showed strong cytoplasmic FASN signals, indicating activation of de novo lipogenesis under steatotic conditions. KPF significantly reduced these fluorescent signals, confirming inhibition of abnormal lipid synthesis at the protein level (*p* = 0.0008, *p* = 0.0001) (Figure 5l,m).

These findings indicate that KPF inhibits liver fat production through regulatory pathways associated with TRIM56, leading to synergistic downregulation of key fat-producing enzymes and effectively preventing liver lipid accumulation.

### 2.5. Silencing the TRIM56 Gene Inhibits the Effect of KPF on NAFLD In Vitro

**Figure 6:**
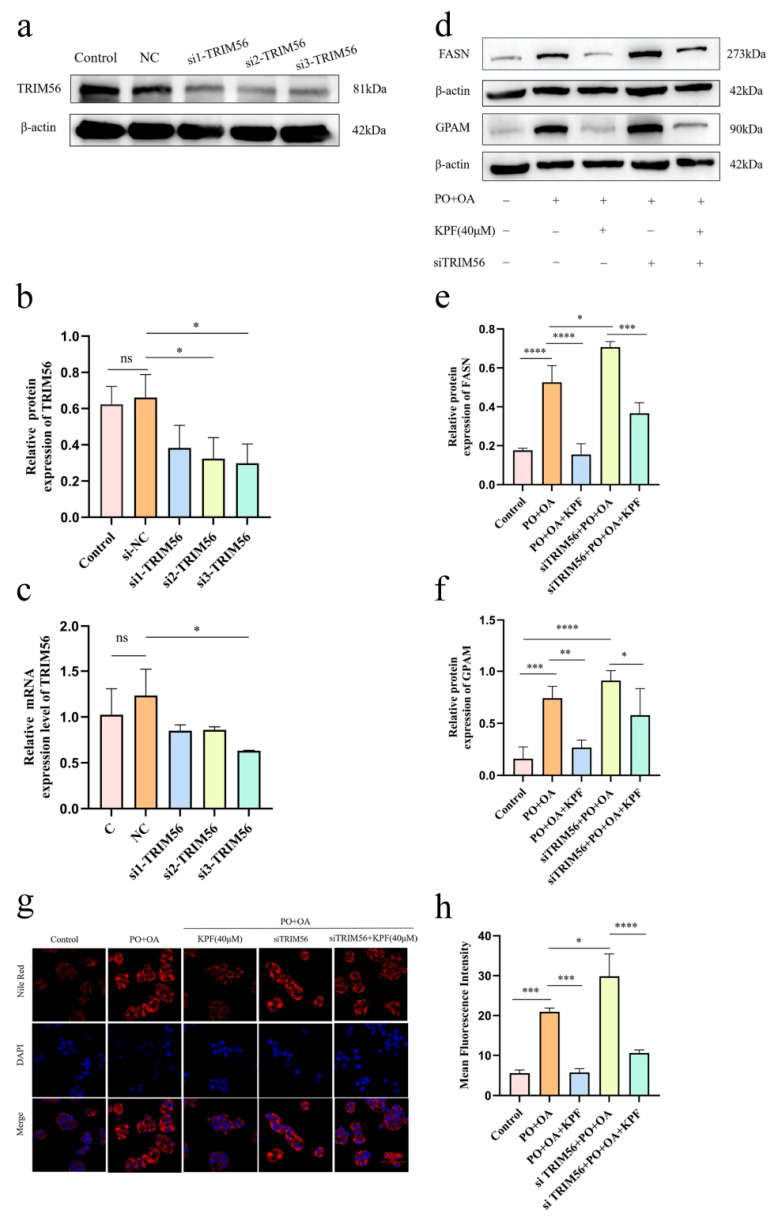
Silencing the TRIM56 gene inhibits the effect of KPF on NAFLD in vitro: (**a**–**c**) qPCR and Western blot detection of siRNA-TRIM56 gene silencing efficiency at the transcriptional and protein expression levels. (**d**–**f**) Western blot detection of the expression levels of key lipid metabolism synthases FASN and GPAM to observe whether knockdown of TRIM56 can weaken the lipid-lowering effect of KPF. (**g**,**h**) Nile red staining to observe lipid accumulation and functional analysis of the effect of TRIM56 knockdown on lipid accumulation. All results are represented as means ± SD (*n* = 3); * *p* < 0.05, ** *p* < 0.01, *** *p* < 0.001, **** *p* < 0.0001. “ns” indicates no statistically significant difference.

To further determine whether TRIM56 is functionally required for the lipid-regulatory effects of KPF, TRIM56 expression was selectively silenced in HepG2 cells using siRNA. Western blot and qPCR analyses confirmed successful gene silencing, as both TRIM56 protein and mRNA levels were significantly reduced compared to control cells (*p* = 0.0311, *p* = 0.0204, *p* = 0.0158) (Figure 6a–c). After TRIM56 suppression, the expression patterns of key lipogenic enzymes were assessed. Western blot results showed significant increases in FASN and GPAM protein levels in cells lacking TRIM56, indicating an upregulated lipogenic state and confirming that TRIM56 loss promotes activation of the lipid synthesis pathway (*p* = 0.0131) (Figure 6d–f).

Nile red staining was used to further evaluate the effect of KPF on lipid accumulation after TRIM56 knockdown. The results showed that, compared with the control group, HepG2 cells lacking TRIM56 exhibited significantly increased lipid droplet accumulation following lipid induction, and the droplet area was larger than that in the control group without TRIM56 knockdown (*p* = 0.0130). After TRIM56 knockdown, the lipid-lowering effect of KPF was significantly reduced but did not disappear entirely (Figure 6g,h). These findings indicate that TRIM56 plays a substantial functional role in mediating the anti-steatotic effects of KPF, while additional TRIM56-independent mechanisms may also contribute to KPF-induced lipid regulation.

## 3. Discussion

NAFLD continues to impose a substantial and high global health challenge, mainly due to its prevalence and the lack of effective drug treatments [4,24]. As the disease advances, individuals face a higher risk of developing NASH, progressive fibrotic remodeling, and hepatocellular carcinoma, all of which pose serious health threats [25,26]. A key process in NAFLD development is hepatic lipid accumulation, which initiates lipotoxicity and metabolic dysfunction; thus, modulation of lipid metabolic pathways is a promising strategy to attenuate disease progression [27]. In the present study, we demonstrate that KPF, a naturally occurring flavonoid, markedly alleviates hepatic steatosis and dyslipidemia in both in vivo and in vitro models of NAFLD. These improvements are accompanied by the restoration of TRIM56 expression and coordinated suppression of lipogenic pathways, supporting a mechanistic link between KPF and lipid metabolic regulation. Although the in vivo HFD model and the in vitro palmitate/oleate-loaded HepG2 model are not identical, they represent complementary levels of NAFLD modeling. The HFD model reflects systemic metabolic disturbances that drive hepatic steatosis, whereas the fatty acid-loading model recapitulates the hepatocellular lipid overload component of this process; therefore, it is suitable for elucidating the mechanism by which KPF mediates TRIM56 signaling in hepatocytes.

Accumulating evidence has identified TRIM family proteins as important regulators of metabolic homeostasis [28]. TRIM56, an E3 ubiquitin ligase, has recently emerged as a key suppressor of hepatic steatosis, with reduced expression observed in NAFLD and hepatocyte-specific upregulation shown to attenuate lipid accumulation [21]. In line with these reports, we observed a significant reduction of TRIM56 expression in HFD-induced NAFLD mice and fatty acid-loaded hepatocytes, whereas KPF treatment consistently restored TRIM56 levels at both the transcriptional and protein levels. These findings suggest that loss of TRIM56 may contribute to NAFLD-associated lipid dysregulation and that KPF counteracts this pathological change.

A major finding of this study is the identification of TRIM56 as a biologically relevant target of KPF. Using molecular docking, molecular dynamics simulations, and cellular thermal shift assays, we demonstrate that KPF engages TRIM56 and stabilizes its protein structure in cells, supporting target engagement under physiological conditions. Although additional biophysical validation is required to precisely determine binding affinity and kinetics, these complementary approaches collectively indicate that KPF engages TRIM56 in cells and may modulate its stability and function.

Previous studies have reported that TRIM56 can interact with FASN and promote its proteasomal turnover, thereby limiting de novo lipogenesis [21]. In the present study, endogenous co-immunoprecipitation confirms that TRIM56 associates with FASN in hepatocytes, supporting the presence of a TRIM56–FASN protein complex relevant to lipid metabolism. Importantly, KPF treatment was accompanied by reduced expression of FASN and other key lipogenic enzymes, including GPAM, DGAT, SCD1, and ELOVL6, suggesting broad suppression of lipogenic programs [29]. While the present data do not directly assess FASN degradation dynamics, the observed association between restored TRIM56 expression and reduced lipogenic enzyme levels is consistent with a TRIM56-centered regulatory mechanism contributing to the inhibition of hepatic lipogenesis.

Loss-of-function experiments further clarified the functional relevance of TRIM56. Silencing TRIM56 enhanced lipogenic enzyme expression and exacerbated intracellular lipid accumulation, confirming its suppressive role in hepatic lipid metabolism. Importantly, TRIM56 knockdown markedly attenuated—but did not completely abolish—the lipid-lowering effects of KPF. This observation indicates that TRIM56 contributes substantially to KPF-mediated lipid regulation, while additional TRIM56-independent pathways may also participate in the overall metabolic response. Such a multi-target mode of action is consistent with the pleiotropic nature of flavonoids and may enhance therapeutic robustness. It should be noted that, although three TRIM56-targeting siRNAs were screened and knockdown efficiency was verified by qPCR and Western blot, potential off-target effects cannot be completely excluded. In addition, rescue experiments were not performed in the present study. Therefore, further studies incorporating rescue strategies are warranted to strengthen the specificity of the observed phenotypic changes.

Despite these advances, several limitations should be acknowledged. First, although computational and cellular assays support target engagement between KPF and TRIM56, future studies employing biophysical techniques such as surface plasmon resonance or isothermal titration calorimetry will be necessary to further characterize binding affinity and kinetics [30]. Although the present study identified the TRIM56–FASN axis as a key pathway involved in the action of KPF, important upstream lipogenic regulators such as SREBP-1c, AMPK, and ACC were not systematically examined. Therefore, the broader regulatory network underlying the anti-steatotic effect of KPF remains to be further clarified. Second, while the present work focuses on TRIM56-dependent mechanisms, KPF may also influence additional signaling pathways involved in lipid metabolism, inflammation, or oxidative stress. Comprehensive omics-based approaches may help identify these complementary mechanisms. Finally, validation of KPF efficacy in additional NAFLD models, including fibrosis-associated or metabolic comorbidity models, will further strengthen its translational relevance.

In summary, our study demonstrates that KPF effectively alleviates hepatic lipid accumulation and metabolic dysregulation in experimental NAFLD. The data support a model in which KPF engages and restores TRIM56, thereby partially regulating downstream lipogenic pathways associated with FASN and related enzymes. This TRIM56-associated regulatory mechanism provides mechanistic insight into the anti-steatotic actions of KPF and identifies TRIM56 as a promising, though not exclusive, therapeutic node for metabolic liver disease.

## 4. Materials and Methods

### 4.1. Experimental Animals and the Development of the NAFLD Model

Sixty male C57BL/6J mice, four weeks of age (approximately 14 ± 2 g in weight), were purchased from Shandong Pengyue Laboratory Animal Technology Co., Ltd. (Jinan, China), a certified supplier of laboratory species. Located at Yantai University. Animals were housed in a controlled, specific-pathogen-free facility equipped with an automated light–dark cycle set to 12 h intervals. Standardized environmental conditions included unrestricted access to sterilized feed and purified water, adequate ventilation, and strict sanitation practices. After one week of acclimatization, the mice were randomly divided into two main groups: a normal control group (Control, *n* = 12) fed a standard chow diet and a model group (*n* = 48) fed a high-fat diet for 8 weeks to induce hepatic steatosis. The detailed composition of the HFD is listed in Supplementary Table S1. After 8 weeks, the HFD-fed mice were further randomly divided into four subgroups (*n* = 12 per group): (1) HFD model group, (2) HFD + rosuvastatin (5 mg/kg), (3) HFD + low-dose KPF (50 mg/kg), and (4) HFD + high-dose KPF (150 mg/kg). The doses of kaempferol (50 and 150 mg/kg) were selected based on previously published in vivo studies showing metabolic and hepatoprotective activity within a comparable dose range, including reports using 50 mg/kg in diabetic/NAFLD-related models and 150 mg/kg in metabolic syndrome models. Rosuvastatin at 5 mg/kg was chosen as a positive control according to prior rodent studies demonstrating efficacy in HFD-associated metabolic disease models [31,32,33,34]. Rosuvastatin was included as an in vivo positive control/reference drug to provide a pharmacological benchmark for lipid-lowering efficacy in the HFD-induced NAFLD model. All the above grouping methods employ computer-based random number generation. The control group continued on the standard diet. All pharmacological agents were freshly prepared and administered by oral gavage once daily for a total of 4 weeks. Body weight, general health indices, and behavioral changes were monitored throughout the experimental period. All animal procedures were performed in accordance with institutional and national guidelines for the care and use of laboratory animals. Appropriate measures were taken to minimize pain, suffering, and distress. Animals were anesthetized during invasive procedures, and analgesics were administered when necessary.

### 4.2. Measurements of Biochemical Parameters

Serum lipid quantification was performed to evaluate metabolic changes associated with NAFLD. The concentrations of TG, TC, and LDL-C were determined using standardized enzymatic colorimetric assays. Commercially prepared kits from Nanjing Jiancheng Bioengineering Institute were used (Nanjing, China), and all procedures were performed strictly according to the operational protocols provided with the reagents.

### 4.3. Histopathological Examination

Liver tissues were immediately immersed in 4% paraformaldehyde (PFA) to preserve morphology and fixed for 24 h. The specimens underwent a progressive dehydration process using a series of ethanol solutions, followed by clearing with xylene prior to being embedded in paraffin wax (Leica Biosystems, Wetzlar, Hesse, Germany). Using a microtome, we then sliced thin tissue sections measuring approximately 5 micrometers in thickness. Three different staining methods were used to evaluate liver pathology: H&E staining to observe overall hepatic structure, inflammatory infiltration, vacuolar changes, and hepatocellular ballooning; Oil Red O staining on frozen liver sections to detect and measure intracellular neutral lipid deposits; and Periodic Acid–Schiff (PAS) staining to visualize glycogen distribution. The process involved oxidation with periodic acid for 10 min, followed by incubation with Schiff’s reagent for approximately 15–20 min in dark conditions. All stained sections were examined under a microscope, and representative images were captured for further analysis.

### 4.4. Cell Culture and In Vitro Lipid Overload Model

The human hepatoblastoma cell line HepG2 was obtained from the American Type Culture Collection (ATCC, HB 8065) (American Type Culture Collection, Manassas, VA, USA). HepG2 cells were originally derived from the liver tissue of a 15-year-old male patient. The cell line is an established human cell line and was used in accordance with institutional guidelines. The HepG2 human liver cancer cell line was maintained in Dulbecco’s modified Eagle medium supplemented with a 10% concentration of fetal bovine serum and a 1% solution of penicillin–streptomycin. Cell cultures were incubated at 37 °C with 5% CO_2_ in a humidified environment. To induce lipid accumulation, an in vitro steatosis model was developed by exposing cells to a 2:1 molar ratio of palmitic acid and oleic acid, resulting in a final concentration of 1 mM. The incubation lasted 24 h. KPF was dissolved in dimethyl sulfoxide (DMSO) at a maximum concentration of 0.1% and used at 10, 20, or 40 µM for another 24 h after lipid induction. KPF was first dissolved in DMSO and then diluted with the vehicle solution before administration. The final concentration of DMSO in the administration solution was maintained at 0.1% or less to minimize the non-specific effects of the solvent. To control for vehicle-related interference, the same vehicle composition and administration volume were used across the relevant experimental groups.

### 4.5. Oil Red O Staining of Cells

Intracellular lipid accumulation was evaluated using a commercial Oil Red O staining kit [35]. HepG2 cells were seeded into culture plates before treatment. Following the completion of the treatments, the culture media were disposed of, and the cells underwent three gentle rinses with PBS. Subsequently, the cells were immobilized at ambient temperature for thirty minutes, followed by two washes with distilled water, and briefly immersed in 60% isopropanol for 20–30 s. The prepared Oil Red O staining solution was added according to the kit protocol. After staining, excess dye was removed by rinsing repeatedly with 60% isopropanol, then washed several times with distilled water until the solution ran clear. Imaging was performed using an inverted fluorescence microscope (Olympus Corporation, Tokyo, Japan), and distilled water was used to keep the cells moist during the imaging process.. Bright-field images were captured with a light microscope, and lipid-stained areas were quantified using ImageJ 1.54f. The proportion of Oil Red O-positive signals was calculated to evaluate lipid accumulation across different treatment groups.

### 4.6. siRNA-Mediated TRIM56 Knockdown

Targeted silencing of TRIM56 was performed using three gene-specific small interfering RNAs (siRNAs) and a non-targeting negative control siRNA, all synthesized by Abmole Bioscience (Shanghai, China). HepG2 cells were seeded one day before transfection and grown to 60–80% confluence. Transfection was carried out using Lipofectamine 2000 (Thermo Fisher Scientific, Waltham, MA, USA) according to the manufacturer’s instructions. Briefly, siRNAs and Lipofectamine 2000 were separately diluted in Opti-MEM reduced-serum medium, incubated for 5 min at room temperature, and then mixed for 20 min to allow for the formation of transfection complexes. The complexes were added to the cells and incubated for 6 h, after which the medium was replaced with complete growth medium. After 48 h of transfection, total RNA and protein were harvested. The knockdown efficiency of the three TRIM56 siRNAs was evaluated, and the siRNA showing the highest silencing efficiency at the protein level was selected for subsequent experiments. TRIM56 knockdown was confirmed by quantitative PCR and Western blot analysis (ECL Gel Imager, Tanon, Shanghai, China).

### 4.7. Protein Isolation and Western Blotting

Liver tissues or HepG2 cells were homogenized in RIPA buffer containing protease and phosphatase inhibitors. Protein samples were homogenized by ultrasonic disruption. Total protein concentration was measured, and equal amounts (20 µg/lane) were loaded onto an SDS-PAGE gel under controlled voltage (80 V for 20 min, followed by 120 V for 90 min). After separation, proteins were transferred onto PVDF membranes [36]. Membranes were briefly blocked and then incubated overnight at 4 °C with primary antibodies recognizing TRIM56, FASN, GPAM, and β-actin. After washing, the secondary antibodies conjugated with horseradish peroxidase were applied for a period of 1.5 h. Following this, enhanced chemiluminescence detection was carried out, and the intensities of the protein bands were measured with the aid of ImageJ software.

### 4.8. Quantitative Real-Time PCR for Gene Expression Profiles

Total RNA was extracted using the SPARK Easy Cell RNA Extraction Kit (AC0205, Spark Jade) (SparkJade, Jinan, China) following the manufacturer’s instructions. The purity and concentration of the extracted RNA were measured with a Thermo Scientific NanoDrop spectrophotometer, Thermo Fisher Scientific, Waltham, MA, USA. Complementary DNA was synthesized using the SPARK Script II ALL-IN-ONE RT SuperMix for qPCR (with gDNA removal; AG0305, Spark Jade). Following the manufacturer’s guidelines, we carried out quantitative real-time PCR with a Roche LightCycler 480 instrument, employing the 2X Universal SYBR Green qPCR Mix (AH0105, Spark Jade) as our reagent of choice (PCR Amplifier, Roche, Basel, Switzerland). Relative transcript levels were calculated using the 2^−ΔΔCT^ method, with *β-actin* as the internal control. Primer sequences for amplification are listed in Table 1, and all primers were synthesized by Qingke Biotechnology Co., Ltd. (Jinan, China).

### 4.9. Immunofluorescence Staining

Cells were cultured in confocal-compatible glass-bottom dishes throughout the experiments. After treatments, samples were fixed in 4% PFA, permeabilized with 0.2% Triton X-100, and blocked with 5% BSA. Primary antibodies targeting TRIM56 and FASN were used and incubated overnight at 4 °C, followed by staining with fluorescent secondary antibodies conjugated to Alexa Fluor dyes [37]. Nuclear staining was performed using DAPI. Imaging was performed using a Leica confocal laser-scanning microscope to visualize protein distribution (Carl Zeiss AG, Oberkochen, Germany). Fluorescence images were acquired using identical confocal settings for all groups within each experiment. DAPI was excited at 405 nm, and the green fluorescence channel was excited at 488 nm. Fluorescence intensity was quantified using ImageJ software after background subtraction. The mean fluorescence intensity (MFI) of each field was calculated and used for statistical analysis.

### 4.10. Nile Red Staining

HepG2 cells were cultured in glass-bottom culture dishes. TRIM56 expression was silenced using the previously described siRNA protocol. To induce lipid overload, cells were then incubated for 24 h with a mixed fatty acid solution containing palmitic acid and oleic acid at a 2:1 molar ratio, reaching a final concentration of 1 mM. After induction, cells were treated with KPF at the specified concentration. Lipid accumulation was visualized with Nile Red staining (Solarbio, Cat. No. G1264), Beijing Solarbio Science & Technology Co., Ltd., Beijing, China [36], and fluorescence images were captured using a Zeiss confocal microscope (Carl Zeiss AG, Oberkochen, Germany). The average fluorescence intensity was quantified to evaluate lipid droplet formation. This procedure assessed the effect of TRIM56 knockdown on lipid synthesis and accumulation after KPF treatment. For Nile Red staining, fluorescence images were obtained under identical imaging settings, and Nile Red was excited at 543 nm. The fluorescence intensity of lipid droplets was quantified using ImageJ software after background correction, and the mean fluorescence intensity was calculated for each image.

### 4.11. Immunoprecipitation

To investigate the potential interaction between TRIM56 and FASN, immunoprecipitation assays were performed. Cells were cultured to a certain quantity and lysed in ice-cold immunoprecipitation lysis buffer supplemented with protease inhibitors. After centrifugation to clarify the cell lysates, they were incubated with anti-FASN antibody or control IgG overnight at 4 °C with gentle rotation. Subsequently, protein A/G agarose beads were added and incubated for an additional 2–4 h. The immunoprecipitated complexes were then extensively washed, released via SDS buffer heating, and then separated by SDS-PAGE and detected through immunoblotting (ECL Gel Imager, Tanon, Shanghai, China).

### 4.12. Molecular Docking and Network Pharmacology Analysis

KPF was used as the primary search term to retrieve its SMILES structure from PubChem. Its potential targets were then predicted using Swiss Target Prediction, CTD, and TCMSP. Genes associated with non-alcoholic fatty liver disease were collected from the GeneCards, DisGeNET, and STRING databases and standardized and converted using UniProt. Targets related to drugs and diseases were compared using Venny 2.1.0 to identify the overlapping targets. KEGG pathway enrichment analysis was then performed for these intersecting genes, with pathways showing significant *p*-values ranked accordingly. The top 30 pathways were visualized through a bubble plot. Molecular docking was performed with AutoDock Vina 1.2.2. The 3D structure of KPF was retrieved from PubChem and energy-minimized [38]. The three-dimensional (3D) model of the receptor protein TRIM56 was generated through homology modeling. Water molecules and nonessential ligands were removed using PyMOL 1.1.2, and AutoDockTools 1.1.2 was used to add hydrogen atoms, calculate charges, and assign AD4 atom types. A docking grid box of 25 × 25 × 25 Å was defined around the predicted active site. Docking poses were ranked based on binding energy (ΔG), with lower ΔG values indicating more stable receptor–ligand interactions. The best docking conformation was visualized with PyMOL 1.1.2 [39].

### 4.13. Molecular Dynamics Simulation

To investigate the TRIM56-KPF complex, we ran molecular dynamics simulations using Gromacs 2025 for a solid 100 nanoseconds. We employed the AMBER14SB force field to parameterize the protein and relied on GAFF2 for the ligand. The entire assembly was then nestled inside a cubic box with a 1.2 nm buffer zone, filled with TIP3P water molecules, and seasoned with 0.15 M NaCl to create a realistic physiological setting while keeping everything electrically neutral. The system was first subjected to energy minimization, followed by equilibration in the NVT and NPT ensembles for a total of 2 ns. The production run was carried out at 310 K and 1 bar for 100 ns with a time step of 2 fs, and the trajectory was saved every 1000 steps. Various metrics were calculated using GROMACS tools, including root-mean-square deviation (RMSD) to assess overall structural stability, root-mean-square fluctuation (RMSF) to analyze residue flexibility, radius of gyration (Rg) to characterize molecular compactness, solvent-accessible surface area (SASA) to reflect solvent exposure, the number of hydrogen bonds between the protein and ligand to evaluate interaction stability, free energy landscape maps (both 2D and 3D), and mmPBSA calculations (average binding free energy and residue decomposition energies).

### 4.14. Statistical Analysis

All data are presented as mean ± SD. For the animal experiments, n represents the number of biologically independent mice in each group. For the in vitro experiments, n represents the number of independent biological experiments performed on separate occasions. Mouse body weight was analyzed using two-way repeated-measures ANOVA, whereas other datasets were analyzed using one-way ANOVA followed by Tukey’s post hoc test for multiple comparisons. Before comparing between groups, use Shapiro–Wilk test to evaluate the normality of data distribution, and the Levene test was used to evaluate the homogeneity of variance. When the assumptions of parameter analysis were met, the differences between multiple groups were analyzed using one-way analysis of variance (ANOVA). Statistical analyses were performed using GraphPad Prism 9. A *p* value < 0.05 was considered statistically significant [23].

## 5. Conclusions

In conclusion, the present study demonstrates that KPF exerts significant hepatoprotective effects against steatosis and dyslipidemia in experimental models of non-alcoholic fatty liver disease. KPF treatment markedly reduces hepatic lipid accumulation, improves systemic lipid profiles, and suppresses aberrant lipogenic programs in both in vivo and in vitro settings.

Mechanistically, our findings support TRIM56 as a biologically relevant molecular interactor contributing to the lipid-lowering effects of KPF. KPF engages TRIM56 and restores its expression under steatotic conditions, and TRIM56 silencing substantially attenuates the ability of KPF to suppress lipogenic enzyme expression and intracellular lipid accumulation. In addition, TRIM56 is shown to associate with FASN in hepatocytes, supporting the involvement of a TRIM56-associated regulatory axis in hepatic lipid metabolism.

Taken together, these results support a model in which KPF alleviates hepatic steatosis, at least in part, through modulation of TRIM56-related lipogenic pathways linked to FASN and other key enzymes. While additional mechanisms may also contribute to the overall metabolic effects of KPF, this study provides mechanistic insight into its anti-steatotic activity and identifies TRIM56 as a promising regulatory node for therapeutic intervention in NAFLD.

## Figures and Tables

**Table 1:** Primer sequences.

Gene	Forward Primers	Reverse Primers
*FASN*	CCTCAGCCGCCATCTACAACATC	GCCAGCGTCTTCCACACTATGC
*GPAM*	TGGCACTGCTGGCAAATGAAGG	CCAGGAGATCACTTCGGGACAGG
*DGAT*	GCGTCCCTCTGCGAATGTTCC	CGATGATGAGCGACAGCCACAC
*SCD1*	TGGGAAGAAGCAAGGGCAAGAAC	ACTGTGTTCAGCAGGGTTTGTGG
*ELOVL6*	GCAATGCTCAGCCCTGGATGTAG	CGAGTGTTCTCCTGTCTGCTTCC
*TRIM56*	ACCTGGTCGTGTCCCTCAGTAAC	CTGCCTCCTTCCTGCCCTCTC
*β-actin*	TGACGTGGACATCCGCAAAG	CTGGAAGGTGGACAGCGAGG

## Data Availability

The original contributions presented in this study are included in the article and Supplementary Materials. Further inquiries can be directed to the corresponding author.

## Supplementary Materials

The following supporting information can be downloaded at: https://www.mdpi.com/article/10.3390/ijms27093767/s1.

## Abbreviations

FASN	Fatty acid synthase
GPAM	Glycerol-3-phosphate acyltransferase, mitochondrial
DGAT	Diacylglycerol O-acyltransferase
SCD1	Stearoyl-CoA desaturase
ELOVL6	Elongation of very long-chain fatty acid protein 6
TC	Total cholesterol
TG	Triglyceride
LDL-C	Low-density lipoprotein cholesterol
DNL	De novo lipogenesis
VLDL	Very low-density lipoprotein
IR	Insulin resistance
TRIM56	Tripartite motif-containing 56
NAFLD	Non-alcoholic fatty liver disease
KPF	Kaempferol
NASH	Non-alcoholic steatohepatitis
HCC	Hepatocellular carcinoma
