# Supplementary material for: Kaempferol Ameliorates Non-Alcoholic Fatty Liver Disease by Targeting TRIM56 to Regulate Lipid Metabolism
Source: Int J Mol Sci. 2026 Apr 23;27(9):3767. doi: 10.3390/ijms27093767 (PMC13163401; doi:10.3390/ijms27093767)
Supplement: Supplementary file 1 [file ijms-27-03767-s001.zip › ijms-4214893-supplementary.pdf]

Supplementary Table S1.

Composition of the High-Fat Diet (HFD) used in this study.

Ingredient	proportion
Basic materials	59%
Lard	18%
Cholesterol	3%
Sucrose	20%
Total	100%
